# A Journal of the Plague Year: Rapid-Response Archiving Meets the Pandemic

**DOI:** 10.1177/1550190620986550

**Published:** 2021-01-27

**Authors:** Mark Tebeau

**Affiliations:** 1 Arizona State University, Tempe, USA

**Keywords:** COVID-19, archive/s, type, collections, digital collections, case study, digital curation, community of practice

## Abstract

Pandemics challenge the foundations of social life, pushing us apart even as we yearn for human contact. A Journal of the Plague Year: An Archive of COVID-19 (JOTPY) emerged as effort by historians and archivists to document the current crisis. Rapid-response archival projects and two decades of digital archiving work, have provided the framework driving the development of JOTPY’s mission, including (among other things) a commitment to addressing the silences in traditional archives, collecting ethically, and developing robust metadata—a particular strength of JOTPY. Nonetheless, archiving pandemic nonetheless presents a distinctive problem that has suggested an altogether new type of rapid-response archive. More broadly, JOTPY is part of a global effort to document the pandemic and seeks to provide a useful model for other research teams involved in this important work.

Pandemics challenge the foundations of social life, pushing us apart even as we yearn for human contact. Disease—Influenza, Cholera, or Ebola—discourages us from those things that had, just days earlier, been the stuff of everyday life: social gatherings, economic exchange, attending school. Accounts of pandemic—whether real or fictitious—underscore our determination to avoid the threat but also the desire for human connections. Archival responses to pandemics and similar health crises reflect a similar impulse: they represent an attempt to collectively makes sense of that which eludes easy comprehension. *A Journal of the Plague Year: An Archive of COVID-19* (JOTPY) emerged in just this context, an effort by historians and archivists to collect and make visible innumerable perspectives that reveal our present and past understandings of science and medicine, culture, art, economics, and social changes wrought by pandemic.

More dramatically, the digital age has changed both how communities both confront and negotiate pandemic. Across the world, schools, families, communities, businesses, and governments have turned to the Internet to confront the disruptions wrought by COVID-19. Schools have turned to distance learning strategies to keep class in session. Families, especially those with aging parents and grandparents, have gathered using video conferencing services. Communities, businesses, and governments have managed communication, continued commercial activities, or maintained constituencies through deeper use of digital tools, including social media, mobile-app driven delivery services, and the Internet. The pandemic has accelerated the movement toward e-commerce and electronic services in ways that may have a profound long-term impact on society. Likewise, archives professionals have drawn on rapid-response archiving—a method of documenting crisis that emerged in the Internet age—in their work to confront and to document the global pandemic.

## Origins of JOTPY

The reality of the pandemic became clear when The World Health Organization declared a global pandemic on March 11, 2020. Two days later, recognizing the moment, as well as the institutional crises attending to teaching during a pandemic, historians at Arizona State University launched JOTPY, seeking for ways to engage students and communities in becoming ad hoc memoirists, collecting digital materials, toward building “a repository of this strange time.”^1^ Born as “A Journal of a Plague Semester,” JOTPY’s formation drew on more than twenty years of work by rapid-response archivists, but also reflected the fundamental naivete about how the scope and scale of the dislocations that would follow from the pandemic.

To date, *JOTPY* has involved universities, museums, and community organizations in collecting; faculty and students in more than 40 courses (university and high school level), with more than 200 individuals from a variety of backgrounds—artists, faculty, archivists, and students—becoming involved in curating the collections, in both small and large ways, dropping into and out of the partnership in a manner that reflected their interests and time constraints. Altogether this team has collected more than 11,000 items through November 30, 2020; *JOTPY* presently consists of thirteen sites (each based in a distinctive item-set), including one Spanish-language effort. The team has used the message-based system Slack (now populated with more than 18,000 communications among our community of practitioners) to engage in discussions about curatorial practices for archives of the pandemic. The team developed collecting protocols, metadata standards, implemented common vocabularies and terms, developed training materials, wrote ethics guidelines, and also designed and developed a theme for Omeka-S that reflected the federated nature of the archive

Rapid-response archival projects, as well as two decades of digital archiving work, have provided the framework driving the development of JOTPY’s mission, including (among other things) a commitment to addressing the silences in traditional archives, collecting ethically, and developing robust metadata. JOTPY was animated by the import of preserving evidence from parts of life—emotions, humor, domestic routines—and from ordinary people—non-elites, members of marginalized communities, the very young and very old—often lost to history.^2^ Not content with such silences, and seeking to do more than merely preserve and amplify the lives of elites, JOTPY has sought those voices that are often muted and distorted by contemporary injustice and inequality. In the United States, for example, people living in poverty, African-Americans, Native Americans, LatinX communities, and the elderly have been among those infected, hospitalized, and killed at higher rates than other groups.^3^ Communities with less social and economic capital also have been disproportionately affected by the pandemic and its economic impacts; these groups also have relatively fewer resources, less access to digital platforms, and less leisure time to document their experiences or participate in the (usually) university-based efforts at documentation.^4^

Addressing silence in an archive is about more than good intentions, it extends to both the practices of how we collect (ethics) and how we present (curate metadata) digital materials. Resources such as *Documenting Ferguson* and *Documenting the Now* reintroduced the importance of ethical collecting as a core value for rapid-response archiving projects and framed these undertakings through the lenses of community as well as the prevalence and immediacy of social media.^5^ More broadly, ethical collecting suggests that collecting in partnership with local communities has emerged as more than just a best practice for taking those stories; it is fundamental to the ethics of rapid-response archival process. Of course, challenges remain, such as how to involve diverse local communities in collecting and curating in a fashion that does not exploit the labor and knowledge of institutional partners or their communities. This has become more complicated during the pandemic as poor and non-white communities have been especially hard hit by the crisis.

Collecting materials without describing them adequately threatens to undo the most ethical of collecting practices. Building rich metadata has not always been a prime feature of rapid-response archives, which have often emphasized collecting stories over fully curating materials in their archives. Viewed over twenty years, one sees the evolution of rapid-response crisis archive. Metadata strategies in these archives has grown more elaborate, as is evident in the evolution of such projects, from the *September 11 Digital Archive* through the *Hurricane Katrina Digital Memory Bank* to *Our Marathon*—in which the metadata is more elaborate, comprehensive, and systemically implemented.^6^ Thus, for the JOTPY team, describing the archive with robust metadata became a fundamental ethic. Without metadata, archives risk becoming attics, their contents perhaps preserved but largely unseen and unused. With metadata, current and future researchers and browsers will be able to aggregate and sort materials across the innumerable dimensions of pandemic experience, making possible far richer historical analysis and policy prescriptions—as well as fostering deeper empathy for past experiences.

The unique nature of pandemics as historical phenomenon also recommended managerial and technical approaches long used in both rapid-responses collecting and public history, including the importance of sharing authority and building iteratively. By definition, pandemics are global events, locally experienced: transmitted through human exchange, intensified or ameliorated through local economies and polities, interpreted and debated through specific secular and sacred traditions. This combination of the intensely local and the undeniably global augments conflict over appropriate responses have heightened the need for diversified collecting—geographically, demographically, and politically. As JOTPY began collecting the project team found itself in conversation with archivists and scholars elsewhere, and we began tentative collaborative work that was bound together by recognizing how sharing authority could accentuate our federated collecting efforts through a common approach to metadata. The curatorial consortium that emerged has since invited and welcomed partners from across the spectrum of galleries, libraries, and museums, involving them to participate on their own terms.^7^ Building an archive demanded a technological approach that was proven and scalable, pushing the team toward Omeka, a well-understand and robust content management system that owes its genesis to rapid-response archiving. Omeka offered the ability to launch the archive quickly—using an inexpensive and hosted version of Omeka through Omeka.net—and to scale the project when (and if) it grew in scope by migrating the initial collection to either Omeka Classic or Omeka-S (multiuser). Eventually, as the project grew in scale and scope, the JOTPY team migrated the site to Omeka-S during the summer of 2020, using the multiuser format to reflect the project’s shared authority, with multiple distinctive sites and item sets within the broader universe of JOTPY (such as the Oral History Initiative or the Southwest Stories effort), balancing the tension between local collecting efforts and the importance of the archives’ global metadata to weave meaning throughout the federated archive.

As important as rapid-response archiving efforts have been to the framing work of JOTPY, the pandemic nonetheless presents a distinctive problem that drove us toward a more iterative approach. Given the lack of clarity of the problem itself, not to mention solutions, documenting the pandemic required that the team collect digital artifacts and build metadata on the fly. In many ways, this makes archives of the pandemic fundamentally different in scope and scale from their peer crisis archives. The current COVID-19 crisis disdains temporal boundaries as it does geographic; as it rolls on, talk of “spikes” and “waves” only heightens our unease about its formlessness and threatened endlessness. It’s true that all crises have long-term repercussions: the Hurricane Katrina Digital Memory Bank, for example, brilliantly documented ongoing economic dislocation as well as the rebuilding of communities and lives. But the COVID-19 pandemic happens and happens and happens some more, no division between event and aftermath yet possible. The pandemic also unmoors us in a second way, obliterating the activities that drew us together and oriented us in time: morning rush hours, work schedules, classroom bells, sports seasons.^8^ This temporal disorientation can turn our faces toward each other: “When will it end?” we ask. And: “do you know what day it is?” But it also threatens to heighten the mistrust incipient in all contagion, as we argue over what stage of the pandemic we have entered and how—even whether— to respond. In short, like the pandemic’s geographic expansiveness, its messy, temporal sprawl threatens division and thus requires collectivity.

In this context, the curatorial team’s instincts to build iteratively and to imagine the archive itself as a community became fundamentally important choices, allowing JOTPY to adapt to an increasingly volatile cultural moment. Indeed, the pandemic both coincided with and drove broader political, economic, and social change. Protest has emerged as a central characteristic of the current crisis, first with demonstrations against mask ordinances and lockdowns. In May, a broader array of national and international protests developed, led by the Black Lives Matter movement in response to the killing of George Floyd by Minneapolis police officers (presently on trial for murder) that was recorded and broadcast on social media. Using the metaphor that racism was itself a virus, the nationwide protests also reflected the social unrest of the pandemic itself, becoming flashpoints for challenging entrenched political regimens tied to race, class, and state-sanctioned violence. The pandemic also emerged in a variety of ways within the broader political sphere. It grew into a potent and central campaign issue in the United State presidential election. At the same time, the multi-year disinformation campaign that became a signal feature of Donald Trump’s presidency—with more than 20,000 documented false or misleading statements according to *The Washington Post*—have complicated the team’s efforts to document the crisis. Broader discussions of truth, hoax, and misinformation—including the question of whether the pandemic even existed—have shaped the archive. In this context—politically polarized and evolving rapidly—the choice to build the project dynamically (rather than to follow a predetermined path) has allowed JOTPY to respond to the multiple and changing dimensions of the pandemic and its cultural expression.

Ironically, JOTPY’s effort to document the pandemic has been fueled through the process itself, which has built a sense of community that has sustained the team. Most critically, that community has provided some respite for the team. This process of community building has allowed the project team to build solidarity that has mitigated against the dislocations of the pandemic and to find support through which they could better confront the stress and trauma associated with both documenting the pandemic and the pandemic itself. At the same time, the pandemic’s uncertainty and dislocations have fueled the administrative development of JOTPY. Somewhat ironically, the loss of more traditional internship opportunities for students—those that meet in person—has led students to find JOTPY and become vital participants in the archive’s curatorial work. Similarly, at Arizona State University, the Humanities Dean, the Institute for Humanities Research, the School of Historical, Philosophical, and Religious Studies, and the Public History Program have supported JOTPY’s development through funding and supporting student (graduate and undergraduate) curators, including funding: a “WPA” program for eight graduate students who had lost in-person summer opportunities, supporting more than a dozen curatorial internships across the university, and underwriting the costs of leading an online history internship course that (to date) has served more than a fifteen other students. These curators, along with their faculty mentors, have noted that curating JOTPY has been more than a professional experience—it has helped them to negotiate the pandemic as a humanitarian crisis even as it has connected them more deeply to their peers.

Moving beyond the work of JOTPY, the work of documenting the pandemic has become a global task; the JOTPY team has delighted in engaging scores of the hundreds of other pandemic archives that have sprung up across the globe. Collectively and individually, these projects will be invaluable to future scholars as well as to archivists thinking about the future of rapid-response archives. Nonetheless, there is much work to be done across the spectrum of pandemic archives, including exploring ways of connecting projects, developing better metadata, and finding ways to express the diversity of human experience of the pandemic across the globe. Similarly, challenges remain for JOTPY, as the team plans its next steps. Important work remains to ensure the archive’s long-term sustainability and to improve users’ ability to discover materials in the archive. Even with extensive metadata, the team remains concerned that JOTPY is not yet friendly enough for users, as well as those who wish to share their story. Also, the collecting continues, especially the quest to implement an ethical approach to documenting the silences of traditional archives. How do we do this better? As JOTPY moves forward, we continue to invite others to join the effort, to help us to document (and to connect through metadata) a diverse range of experiences of the pandemic. Reach out to us via email at covid19archive@asu.edu.

## Notes

1. Catherine O’Donnell, “Journal of a Plague Semester,” March 13, 2020, https://covid-19archive.org/s/archive/item/12321.

2. Public historians use Michel-Rolph Trouillot, *Silencing The Past: Power and the Production of History* (New York: Beacon Press, 2015) to underscore the consequences of archival and interpretive silence. In thinking about archival silences, see for example, Rodney G.S. Carter, “Of Things Said and Unsaid: Power, Archival Silences, and Power in Silence,” *Archivaria*, September 25, 2006; David Thomas, Simon Fowler, Valerie Johnson, editors, The Silence of the Archive (London: Facet Publishing, 2017).

3. Examples of this are many. See, for instance, Don Bambino, et. al., “Disproportionate Impact of COVID-19COVID-19 on Racial and Ethnic Minorities in the United States,” *Clinical Infectious Diseases*, June 20, 2020, https://doi.org/10.1093/cid/ciaa815; Grace A. Noppert, “COVID-19COVID-19 is hitting black and poor communities the hardest, underscoring fault lines in access and care for those on margins,” *The Conversation*, April 9, 2020. Anna North, “The coronavirus pandemic exposes America’s devastating inequalities,” *Vox*, April 10, 2020.

4. On the class and racial divide made stark by the pandemic there is much work. See, for instance, Jennifer Valentino-DeVries, Denise Lu, and Gabriel J.X. Dance, Location Data Says It All: Staying at Home During Coronavirus Is a Luxury, *New York Times*, April 3, 2020.

5. The critical work of ethical collecting through partnership with local communities is emphasized by Documenting the Now, especially its white paper Bergis Jules, Ed Summers, Dr. Vernon Mitchell, Jr., “Ethical Considerations for Archiving Social Media Content Generated by Contemporary Social Movements: Challenges, Opportunities, and Recommendations, April 2018. Notably, the work of the Mellon Foundation offers a definition of community-based archives “as collections of materials gathered, collected, and shared primarily by members of a marginalized community to document their collective histories,” in an effort to combat “symbolic annihilation” of marginalized communities from mainstream memory institutions, and in recognizing that they are essential to the creation of a more inclusive and polyvocal American story.” webcache: Mellon Foundation Call for Proposals, June 28, 2020 <accessed June 28, 2020>. Following this model, JOTPY has partnered with more than a dozen Native American, LatinX, and religious communities composed of and working with hard-hit LatinX migrants, undocumented workers, and Native American communities to meet JOTPY’s mission of trying to build an archive that takes account of the diversity of the pandemic experience.

6. On the importance of metadata in making disaster archives legible to publics see Sheila Brennan, “What’s Next for Digital Memory Banks?,” *Lot 49*, May 6, 2013.

7. The concept and its application were originally developed in the context of oral history by Michael Frisch, *A Shared Authority: Essays on the Craft and Meaning of Oral and Public History* (New York, SUNY Press, 1990; also see, for example, Bill Adair, Benjamin Filene, Laura Koloski, editors, *Letting Go?: Sharing Historical Authority in a User-Generated World* (New York: Routledge, 2011).

8. Elana Gomel writes that “The responses of pandemic- stricken societies follow a common pattern. . .. This pattern comprises panic, dissolution of socioeconomic structures, and despair, succeeded by a makeshift return to normality once the disease has run its course.” Elana Gomel, “The Plague of Utopias: Pestilence and the Apocalyptic Body” *Twentieth Century Literature* 46, no. 4 (2000): 405–33, at 408. COVID-19 displays that pattern, with the “return to normality” disrupted in the United States by rapid resurgence of disease and a threatened, though debated, recurrence of the initial sequence.

